# New Salt of Mercury and Quinia

**Published:** 1844-07-27

**Authors:** 


### NEW SALT OF MERCURY AND QUINIA.

The combination of oxymuriate of mercury and tincture of bark has been long known as a remedy for the treatment of scrofula and enlarged mesenteric glands, also in the treatment of strumous ophthalmia. This combination is well known to be unchemical, the salts being decomposed by the bark. Mr. R. N. M'Dermot, of Dublin, convinced of the value of a combination of the active principle of the barks with salt of mercury—"a combination which, according to the concurring testimony of various physicians, accelerates, in a remarkable manner, the constitutional action of mercurials, was brought to think that a definite compound might be formed in which the bichloride would perform the part of an acid, and the alkaloid quinia form the base, and which would combine the therapeutic value of these two important substances." On trial he found the results were exactly as he had anticipated. He obtained a double salt, a proto-chloride of mercury and quinia, chemically combined. On subjecting it to the strictest analysis, no trace of bichloride could be detected. The intimate combination of the active principle of the bark with mercury in the form just indicated, will, in his opinion, render it less liable to produce the ill effects of mercury on some constitutions, while its efficacy as a general remedy must be much enhanced. He anticipates that the combination of these two agents will rarely fail of producing a happy result in the diseases of the eye generally, but especially when scrofula is present.—*Dublin Medical Press.*

